# Supplementary material for: Quantitative single-protein imaging reveals molecular complex formation of integrin, talin, and kindlin during cell adhesion
Source: Nat Commun. 2021 Feb 10;12:919. doi: 10.1038/s41467-021-21142-2 (PMC7876120; doi:10.1038/s41467-021-21142-2)
Supplement: Supplementary file 2 — Supplementary Information [file 41467_2021_21142_MOESM2_ESM.pdf]

Quantitative single-protein imaging reveals molecular complex formation of integrin, talin, and kindlin during cell adhesion

Lisa S. Fischer[1,2, †], Christoph Klingner[1,2, †], Thomas Schlichthaerle[3,4, †], Maximilian T. Strauss[3,4,5], Ralph Böttcher[6], Reinhard Fässler[6,*], Ralf Jungmann[3,4,*] and Carsten Grashoff[1,2,*]

[1]Department of Quantitative Cell Biology, Institute of Molecular Cell Biology, University of Münster, Münster D-48149, Germany.

[2]Group of Molecular Mechanotransduction, Max Planck Institute of Biochemistry, Martinsried D-82152, Germany.

[3]Faculty of Physics and Center for Nanoscience, LMU Munich, Munich D-80539, Germany.

[4]Research Group Molecular Imaging and Bionanotechnology, Max Planck Institute of Biochemistry, Martinsried D-82152, Germany.

[5]Department of Proteomics and Signal Transduction, Max Planck Institute of Biochemistry, Martinsried D-82152, Germany.

[6]Department of Molecular Medicine, Max Planck Institute of Biochemistry, Martinsried D-82152, Germany.

[†]These authors contributed equally: Lisa S. Fischer, Christoph Klingner, Thomas Schlichthaerle.

[*]Correspondence to C.G. (grashoff@wwu.de), R.J. (jungmann@biochem.mpg.de) or R.F. (faessler@biochem.mpg.de).

Supplementary Information

Supplementary Figure 1-11

Supplementary Table 1-7

Supplementary Figures

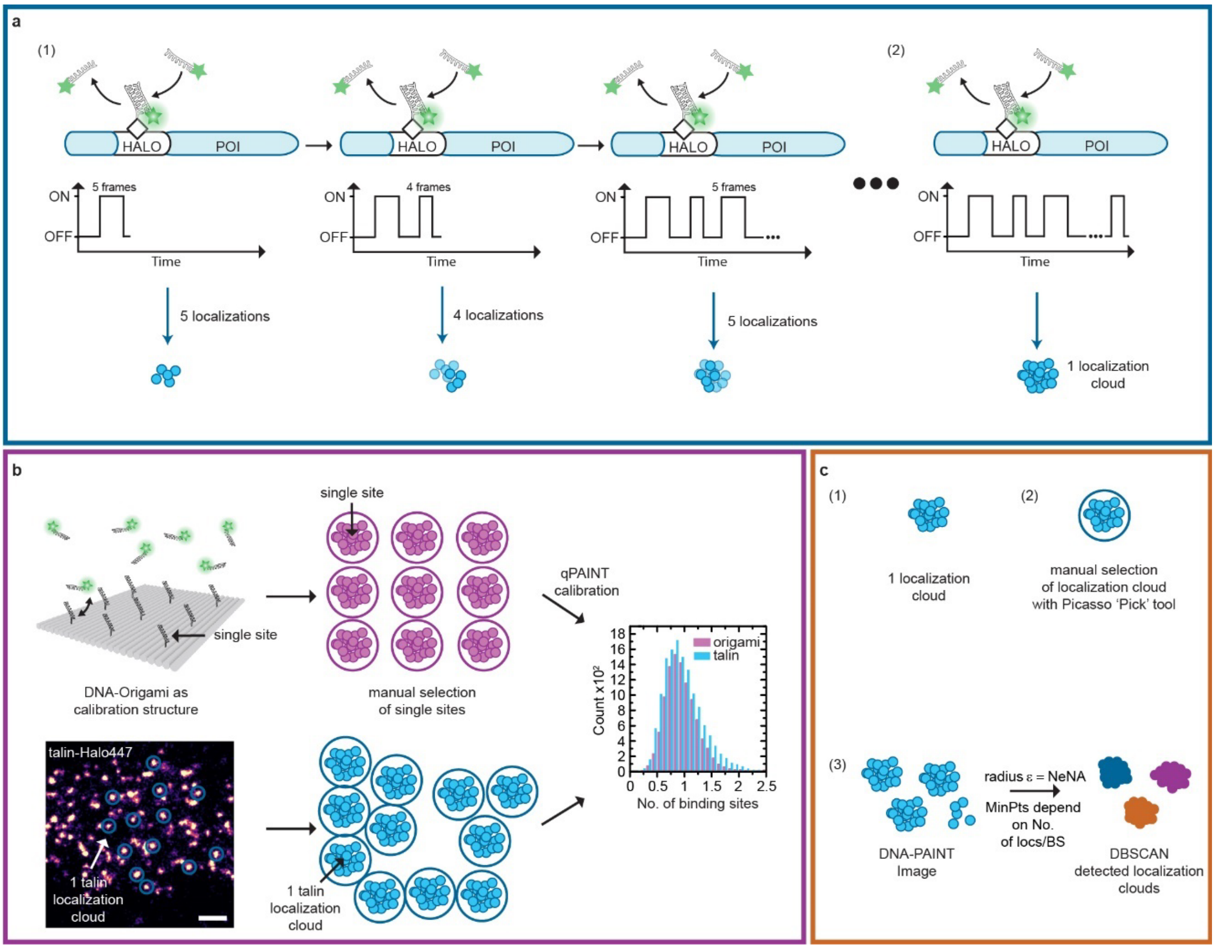

Supplementary Figure 1 Overview of experimental procedures and terminology. (**a**) The accumulated signal caused by the repetitive binding of an imager strand to the protein of interest (POI) labelled with a docking strand is defined as a localization cloud. (**b**) DNA origami with predefined number of binding sites are imaged together with cells and used for calibration. By manually selecting and analyzing single-binding sites on DNA origami, influx rates can be extracted to determine the number of molecules in talin localization clouds. (**c**) Visual description of important terminology used in this manuscript; nearest neighbor analysis (NeNA), minimal points (MinPts), localizations per binding site (locs/BS). Scale bar: 110 nm (**b**).

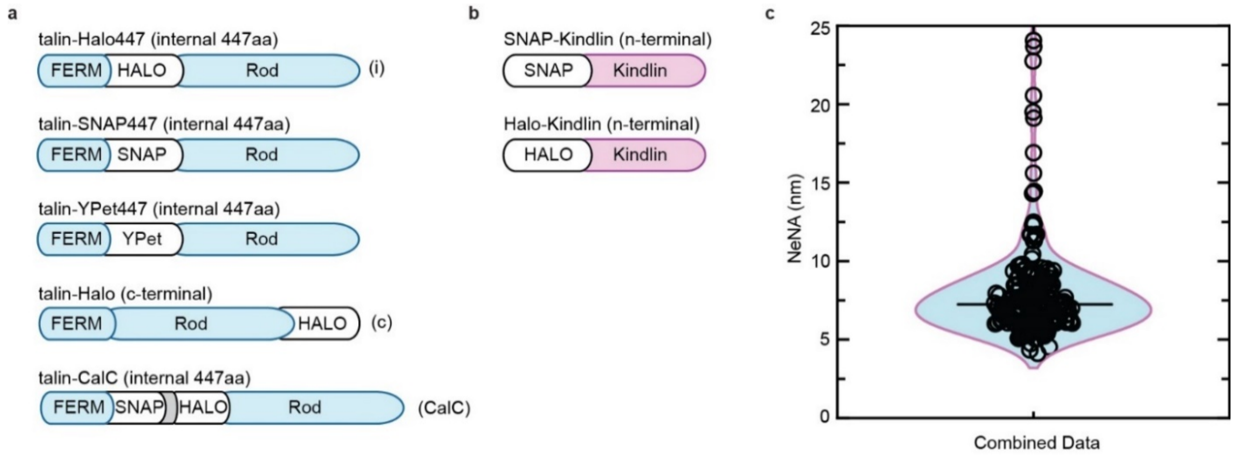

Supplementary Figure 2 Overview of expression constructs. (**a**) Talin-1 was internally tagged by inserting a HaloTag, a SNAP-tag, or a YPet-tag after the N-terminal FERM domain at amino acid (aa) 447. To control for effects of differential tag insertion sites, talin-1 was C-terminally fused with the HaloTag. To generate a positive control for spatial proximity, a SNAP–HaloTag cassette was inserted into talin-1 at aa 447; both tags are separated by seven amino acids (linker peptide: GPGGAGP). (**b**) For kindlin labelling, SNAP-tag or HaloTag was fused N-terminally to kindlin-2. (**c**) Calculation of the overall localization precision by nearest neighbor-based analysis (NeNA) reveals an average localization precision of about 7 nm considering all experiments (n = 209 cells). Median is indicated. Source data are provided in the Source Data file.

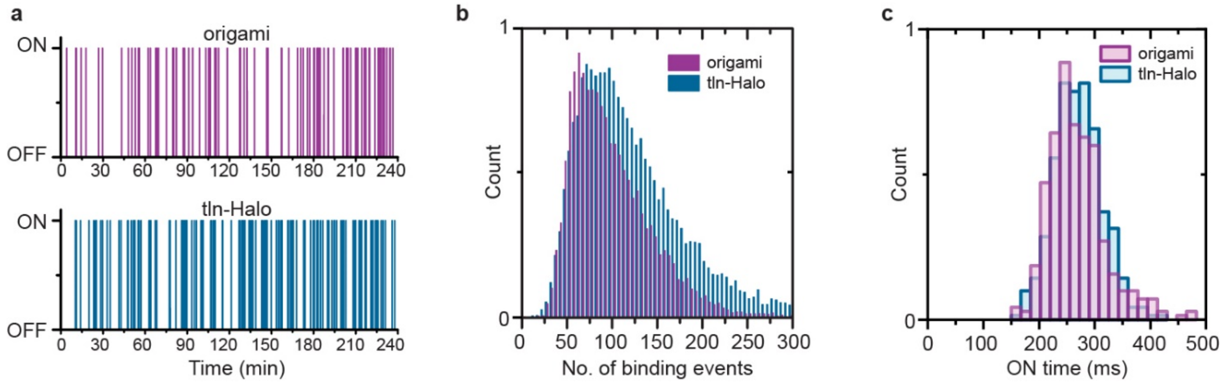

Supplementary Figure 3 Comparison of qPAINT associated with DNA-Origami and talin-Halo localization clouds. (**a**) Representative binding event histories of a single DNA origami (origami) binding site (top) and a talin-Halo447 (tln-Halo) localization cloud (below). (**b**) Statistical analysis of binding events on DNA origami were used to calibrate the mean number of binding events per docking site. Subsequent comparison to the binding numbers per localization cloud reveals that a talin localization cloud contains one docking strand and thus one talin-1 protein ($n_{Origami}$ = 9936; $n_{tln\text{-}Halo.}$ = 13457 localization clouds). (**c**) Analysis of the on-time of single binding events on DNA-origami and tln-Halo localization clouds reveals similar values ($n_{Origami}$= 359; $n_{tln\text{-}Halo.}$ = 359 localization clouds). Source data are provided in the Source Data file.

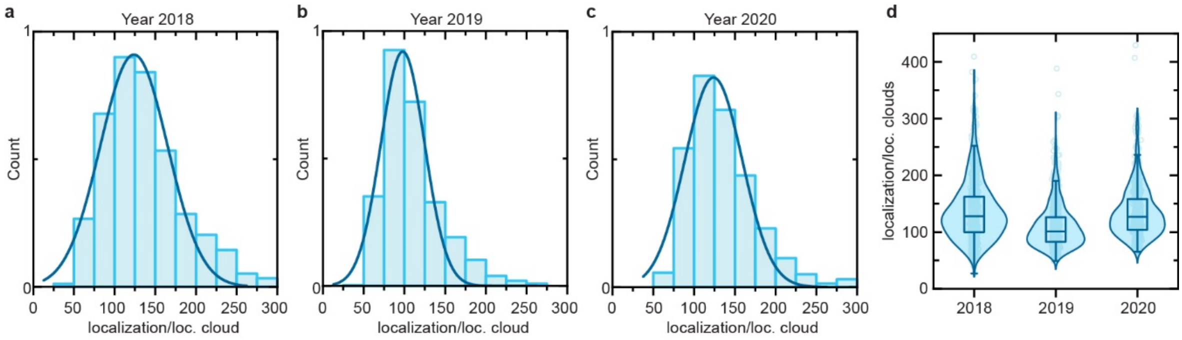

Supplementary Figure 4 qPAINT reproducibility and evaluation of DBSCAN parameter MinPts. (**a-c**) Histograms of localizations per localization cloud (loc. cloud) reveal unimodal distributions and highly similar values over three years of data acquisition. (**d**) Boxplots of localization per localization cloud reveal similar mean values for all data sets imaged over three years ($median_{2018}$ = 128; $median_{2019}$ = 101; $median_{2020}$ = 127) (n_{2018} = 1076; n_{2019} = 947; n_{2020} = 903 localization clouds). Boxplots show median and 25th and 75th percentage with whiskers reaching to the last data point within 1.5x interquartile range. Source data are provided in the Source Data file.

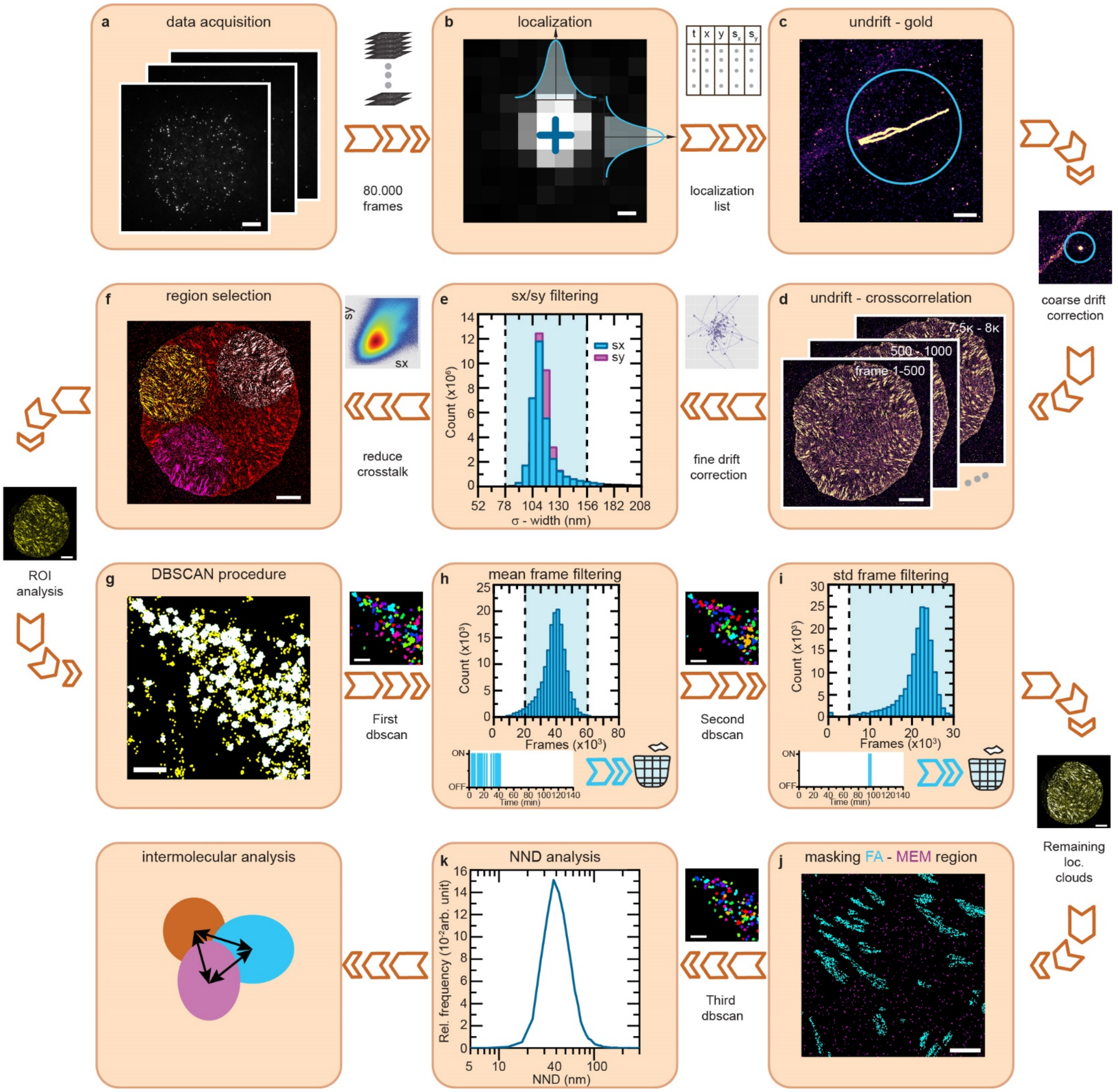

Supplementary Figure 5 Workflow of data acquisition and data processing. (**a**) Data were acquired using image acquisition parameters described in the Methods section and Supplementary Tables. (**b**) Images were reconstructed by identification and fitting of single-molecule spots in each frame. (**c**) Images were coarse drift corrected using 5–10 gold particles as fiducial markers. (**d**) Subsequently, images were drift-corrected using image sub-stack

redundant cross-correlation (RCC). (**e**) In an additional post-processing step, the sigma value (sx/sy) derived from the gaussian localization fit in **b**, was filtered to eliminate double binding and spurious binding events. (**f**) Next, regions of interest (ROI) were manually selected and used for further nearest neighbor distance (NND) analysis. (**g**) DBSCAN analysis was used to detect distinct localization clouds in ROIs. (**h**) Repetitive transient binding to single sites in the cells leads to a mean frame of approximately half the number of total frames in the acquisition window. Therefore, mean frame filtering can be used to remove DBSCAN detected localization clouds, which are not continuously visited by an imager strand over the whole course of image acquisition. (**i**) Filtering of the standard deviation frame removes long but non-repetitive binding events (imager sticking) indicating unspecific binding events. Such events occur randomly during the time of acquisition and the mean frame is typically located within the frames of the long non-repetitive binding event. As a result, the standard deviation is small, which can be used to isolate these non-specific binding events[44]. (**j**) Gaussian blur-based masking was performed to discriminate between focal adhesion (FA) and free membrane regions (MEM). (**k**) The center of mass for each individual localization cloud (shown as relative frequency) was calculated to allow for NND analysis using a k-dimensional tree algorithm. Scale bars: 10 µm (**a**), 130 nm (**b**), 500 nm (**c**), 10 µm (**d**), 10 µm (**f**), 200 nm (**g**), 1.5 µm (**j**).

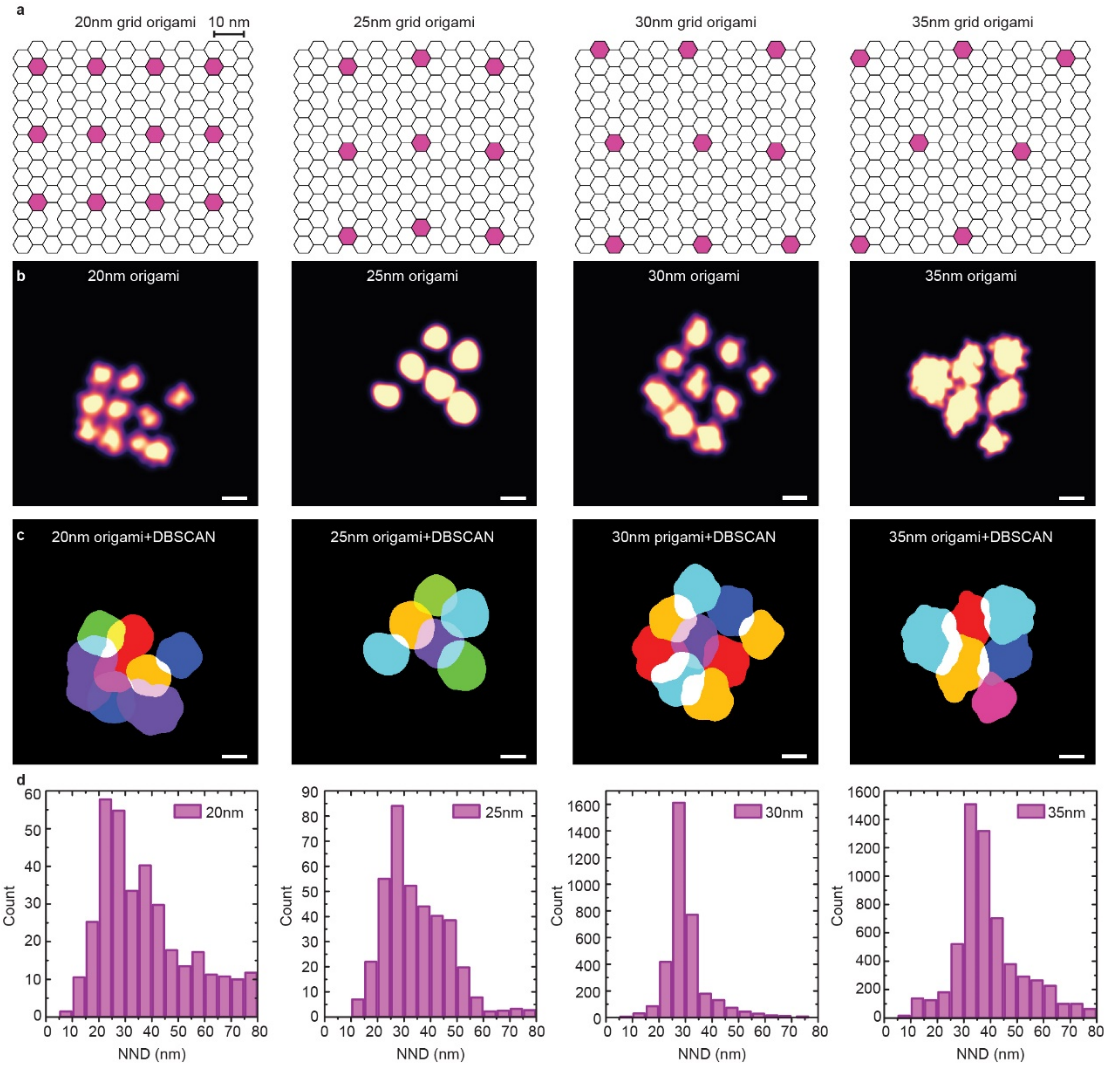

Supplementary Figure 6 Analysis of DBSCAN resolution limit. (**a**) Design schematics of four different DNA origami with twelve binding sites spaced 20 nm, nine sites spaced 25 nm or 30 nm, and seven binding sites spaced 35 nm apart. (**b**) DNA-PAINT images of DNA origami structures shown in (**a**). (**c**) DBSCAN analysis of DNA origami in (**b**) with parameter sets used for FA analysis reveals efficient separation of binding sites down to 25 nm and merging of individual binding sites at 20 nm distances. Visualization is based on the convex hull of grouped localization clouds. (**d**) Nearest neighbor distance (NND) histograms depict the faithful binding site detection by fully automated DBSCAN analysis down to 25 nm (n_{20nm}

= 4092; n_{25nm} = 4113; n_{30nm} = 3901; n_{35nm} = 7251 NN distances). Scale bars: 5 nm (Hexagon (**a**)), 20 nm (**b**, **c**). Source data are provided in the Source Data file.

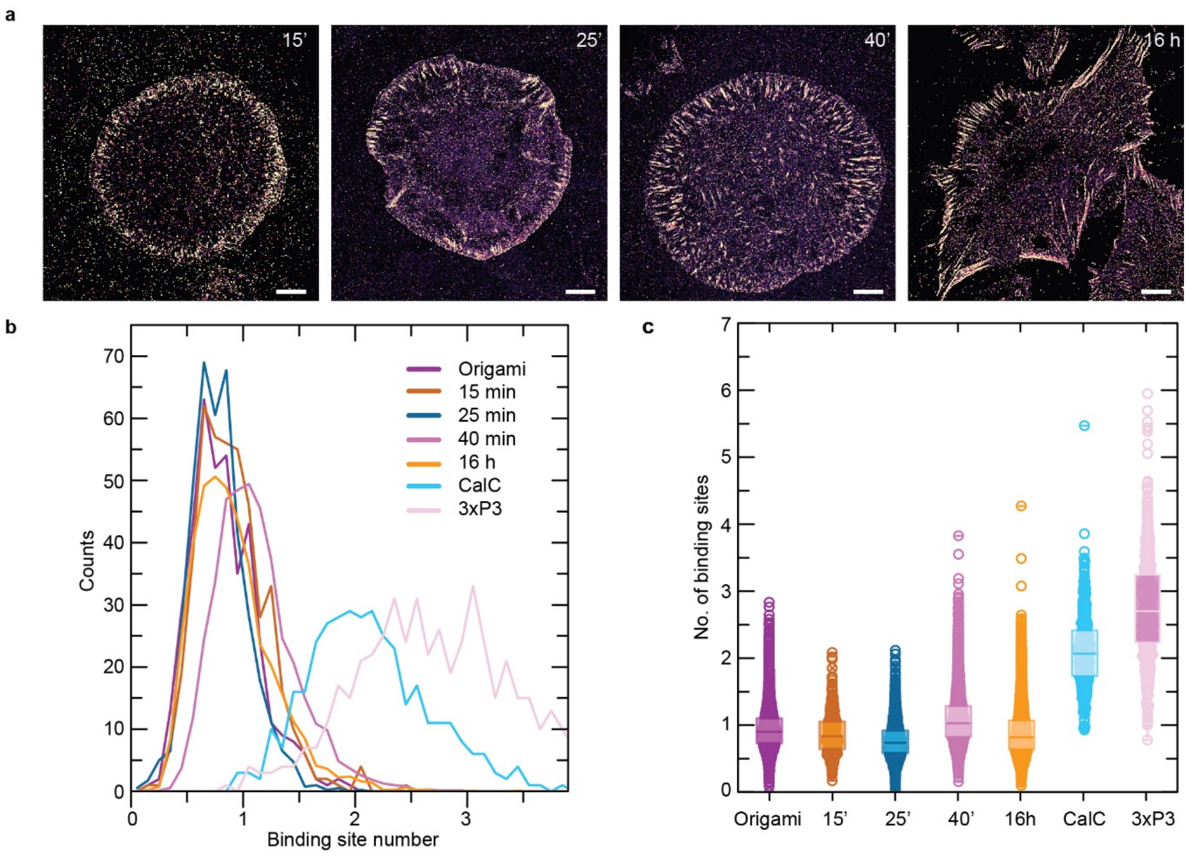

Supplementary Figure 7 Time-resolved qPAINT analysis of talin-1 localization clouds. (**a**) DNA origami (not resolved at this magnification) were seeded next to cells and analyzed in parallel to allow qPAINT analysis at distinct time points during cell spreading. (**b**) qPAINT analysis reveals single binding sites per talin localization cloud at each time point, whereas double labelling of the calibration control (CalC) construct and 3xP3 origami structures (see Figure 1) show the expected two and three binding sites per localization cloud. (**c**) Boxplots of qPAINT analysis reveal medians around one binding site for talin-Halo447 data, two binding sites for CalC, and three binding sites for 3xP3 origami data. Together, this demonstrates that talin localization clouds contain one talin protein at all time points ($n_{Origami}$ = 24; $n_{15'}$ = 1; $n_{25'}$ = 4; $n_{40'}$ = 7; n_{16h} = 8; n_{CalC} = 3; n_{3xP3} = 1 cells). Boxplots show median and 25th and 75th percentage with whiskers reaching to the last data point within 1.5x interquartile range. Scale

bars: 10 μm (at 15’), 7 μm (at 16h), 5 μm (at 25’, 40’). Source data are provided in the Source Data file.

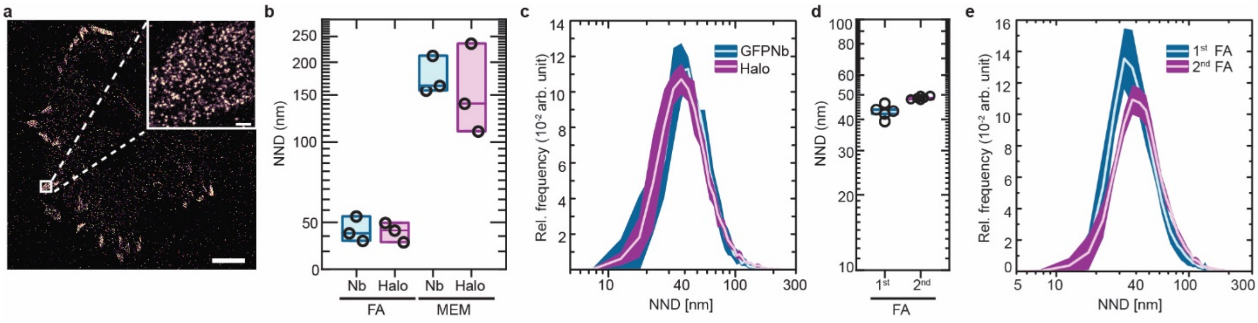

Supplementary Figure 8 Comparison of talin-1 visualized by GFP-Nanobody and HaloTag. (**a**) Representative GFP-Nanobody-based DNA-PAINT image of talin-YPet447 cells and zoom-in revealing talin-1 molecules that are indistinguishable from those observed in labelled talin-Halo447 cells. (**b**) Nearest neighbor distance (NND) analysis of GFP-Nanobody (Nb) and HaloTag (Halo) in focal adhesion (FA) and free membrane regions (MEM) demonstrates that results are independent of the labelling tag and labelling protocol (n = 3 cells). (**c**) NND distributions shown as relative frequency (Rel. frequency) in FAs from GFP-Nanobody- and HaloTag-labelled cells (n = 3 cells). (**d**) Talin NND analysis (in FA) depicting effects of an Exchange-PAINT experiment on the determined intermolecular distances. NNDs slightly shift towards higher values in FAs for the second Exchange-PAINT data set (n = 5 cells). (**e**) Distance distribution curves indicate slight systematic changes towards higher NNDs for the second round of Exchange-PAINT experiment. Overall, the systematic error inherent to Exchange-PAINT experiments seems negligible with regard to the here described biological effects (n = 5). Boxplots show median and 25th and 75th percentage with whiskers reaching to the last data point within 1.5x interquartile range. NND distributions show mean (line) ± SD (shaded area). Scale bars: 6 µm (**a**), 200 nm (**a** inset). Source data are provided in the Source Data file.

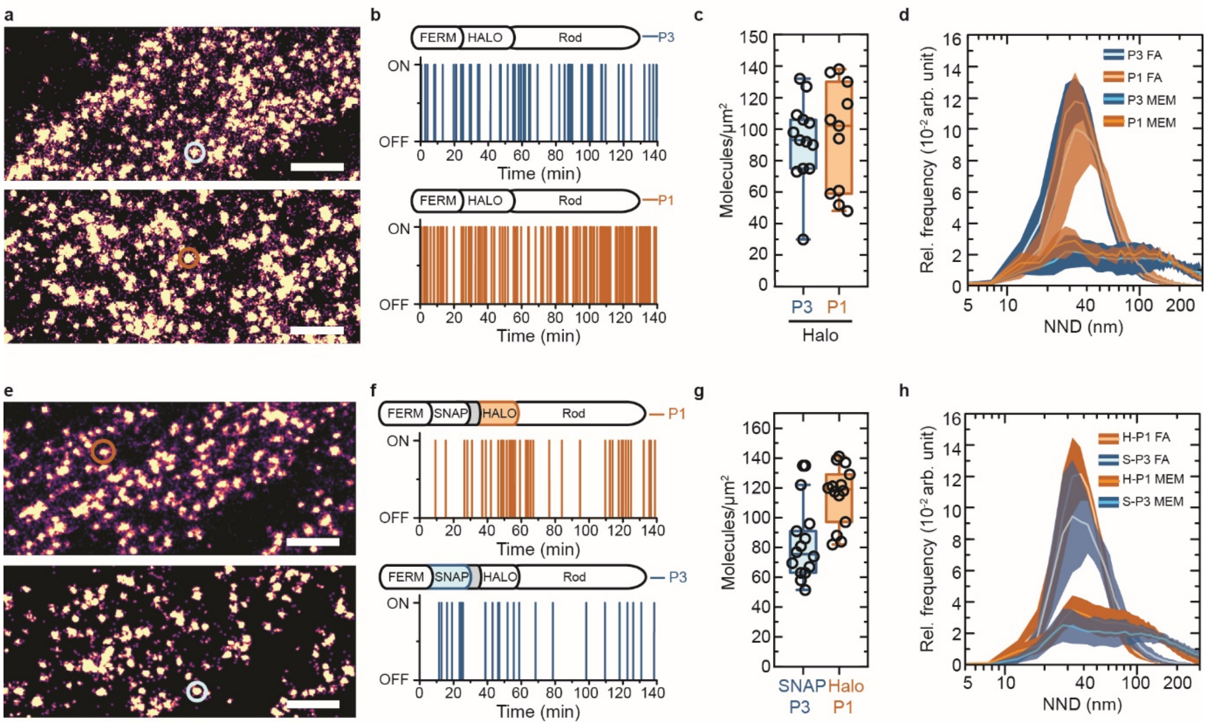

Supplementary Figure 9 Controls for SNAP-tag/HaloTag Exchange-DNA-PAINT experiments. (**a**) To control labelling efficiency of different DNA sequences, DNA-PAINT images of talin-Halo447 cells were acquired using P3 or P1 imager strands. (**b**) Comparison of P1 (orange) and P3 (blue) binding histories. (**c**) After accounting for the different imager strand labelling efficiencies, the detected molecular densities are indistinguishable using P1 and P3 strands. (n_{P3} = 13; n_{P1} = 11 cells). (**d**) Distribution curves of the nearest neighbor distance (NND) shown as relative frequency (Rel. frequency) in focal adhesion (FA) and free membrane regions (MEM) using P1 and P3 are consistent (n_{P3} = 13; n_{P1} = 11 cells). (**e**) To test for the labelling efficiency of HaloTag and SNAP-tag, talin-CalC expressing cells were labelled with either SNAP- or HaloTag and subjected to DNA-PAINT imaging. (**f**) Traces of HaloTag labelled with P1 (orange) and SNAP labelled with P3 (blue). (**g**) Analysis of the molecular density in focal adhesions (FAs) by Exchange-DNA-PAINT reveals an approximately 30 % less efficient labelling of the SNAP-tag (n_{CalC} = 14 cells). (**h**) NND distribution curve of SNAP

(S)- and HaloTag (H) in FAs and MEM reveal a lower frequency count for SNAP-tag leading to a slightly decreased number of detected molecules (n_{CalC} = 14 cells). Boxplots show median and 25th and 75th percentage with whiskers reaching to the last data point within 1.5x interquartile range. NND distributions show mean (line) ± SD (shaded area). Scale bars: 200 nm (**a**, **e**). Source data are provided in the Source Data file.

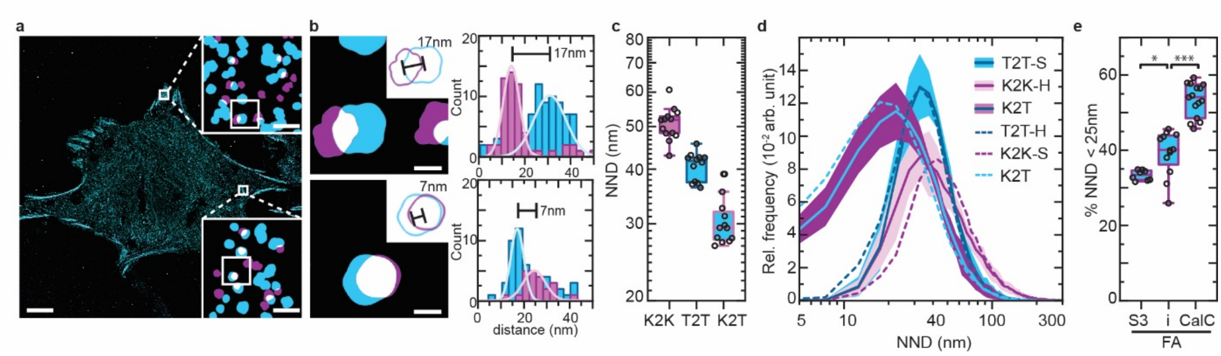

Supplementary Figure 10 Validation of talin-1 and kindlin-2 molecules undergoing spatial association in FAs. (**a**) Representative image of a reconstituted cell with labelled talin-SNAP447 and Halo-kindlin molecules. (**b**) Zoom into focal adhesions (FAs) reveals talin-1 and kindlin-2 molecules in close spatial proximity. Gaussian fits of the aligned single-molecule localizations to their center-of-mass reveal neighboring talin-1 (blue) and kindlin-2 (purple) molecules at distances of 17 nm (σ_{Peak1}= 3.8nm; σ_{Peak2}=7.0nm) and 7 nm (σ_{Peak1}= 2.9nm; σ_{Peak2}=4.8nm). (**c**) Nearest neighbor distance (NND) analyses reveal the molecular spacing between kindlin-2 (K2K) and talin-1 molecules (T2T). The average kindlin-to-talin distances (K2T) are significantly lower (n = 12 cells). (**d**) Talin-SNAP447 (T2T-S), Halo-kindlin (K2K-H), and K2T NND distributions shown as relative frequency (Rel. frequency) indicate a shift of K2T towards shorter distances. Dashed lines show data from Fig. 3d with Talin-Halo447 (T2T-H), SNAP-kindlin (K2K-S) and K2T NND distributions. NND distributions show mean (line) ± SD (shaded area). (**e**) Simulations of randomly spaced molecules (S3) using the experimentally observed densities indicate that 35 % of talin-1 and kindlin-2 molecules can be expected in close spatial proximity (<25 nm) merely because of high protein density in FAs. By contrast, 42 % of labelled kindlin molecules are in close proximity to the next internally tagged talin-molecule in experimental data sets, suggesting specific association (i). Experiments of talin-CalC (same data set as in Fig. 3e), which is a mimic of perfect spatial proximity, yields a value of 55 % (CalC) (n_{S3} = 8; n_i = 12; n_{CalC} = 15 cells) ($p_{S3\ vs\ i}$ = 0.01553;

$p_{i\ vs\ CalC} = 1.4*10^{-7}$). Boxplots show median and 25th and 75th percentage with whiskers reaching to the last data point within 1.5x interquartile range. Two-sample t-test: *** $p \leq 0.001$, * $p \leq 0.05$. Visualization of (**a**) and (**b**) is based on the convex hull of the grouped localization clouds. Scale bars: 5 µm (**a**), 100 nm (**a** insets), 20 nm (**b**). Source data are provided in the Source Data file.

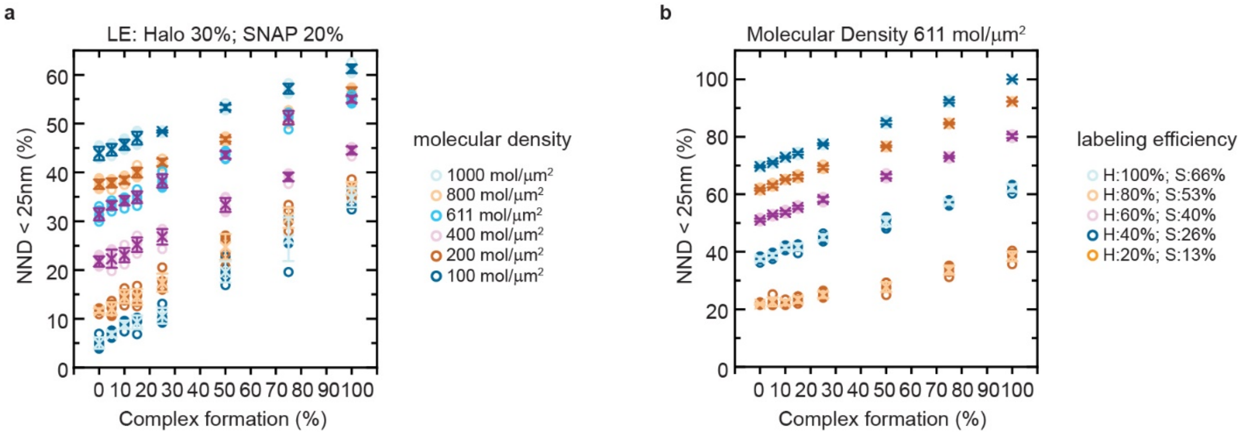

Supplementary Figure 11 Theoretical simulations of colocalization experiments assuming different molecular densities and labelling efficiencies. (**a**) The percentage of binding sites closer than 25 nm (nearest neighbor distance (NND) < 25nm) was plotted over the percentage of complex formation. Different molecular densities were assumed covering a range from 100–1,000 molecules/μm^2 per protein population with the same labelling efficiencies (LE) for SNAP- and HaloTag (S: 20 %; H: 30 %). For each complex formation at the given molecular density five independent data sets were simulated. (**b**) Different LE's were assumed for HaloTag (H) from 20–100 % LE and SNAP-tag (S) from 13–66 %, showing how the range of co-localization depends on the LE at a fixed molecular density. For each complex formation at the given labelling efficiencies five independent data sets were simulated (n = 5). Crosses indicate the mean of the data, error bars the standard deviation. Source data are provided in the Source Data file.

Tables

Supplementary Table 1 | Ligands with conjugated DNA-PAINT docking sequences.

Docking strand	Sequence	5'-mod	3'-mod	Company
CA-P1	TTA TAC ATC TAT T	Chloroalkane	Atto488	Biomers
CA-P3	TTT CTT CAT TAT T	Chloroalkane	Atto488	Biomers
CA-R1	TTT CCT CCT CCT CCT CCT CCT	Chloroalkane	None	Biomers
BG-R2	AAA CCA CCA CCA CCA CCA CCA AA	Benzylguanine	Atto488	Biomers
BG-P1	TTA TAC ATC TAT T	Benzylguanine	Atto488	Biomers
BG-P3	TTT CTT CAT TAT T	Benzylguanine	Atto488	Biomers
BG-P5	TTT CAA TGT AT	Benzylguanine	Atto488	Biomers
TZ-P3	TTT CTT CAT TA	Tetrazine (Methyltetrazin-PEG5)	None	Biomers
Azide-P3	TTT CTT CAT TA	Azide	None	Biomers

Supplementary Table 2 | DNA-PAINT imager sequences.

Imager strand	Sequence	5'-mod	3'-mod	Company
P1	AGA TGT AT	None	Cy3b	Eurofins
P3	AAT GAA GA	None	Cy3b	Eurofins
P5	TAC ATT GA	None	Cy3b	Eurofins
R1	AGGAGGA	None	Cy3b	Metabion
R2	TGGTGGT	None	Cy3b	Metabion

Supplementary Table 3 | Folding protocol.

Component	Initial conc. [µM]	Parts	Pool conc. [nM]	Target conc. [nM]	Vol. [ul]	Excess
Scaffold	0.1	1	100	10	4	1
Core Mix	100	164	609.76	100	6.56	10
P3 Mix	100	12	8333.3	1000	4.8	100
Biotin 1:10	100	80	1250	10	0.32	1
H2O					20.32	
10x Folding Buffer					4	
Total Vol.					40	

Supplementary Table 4 | Imaging parameter.

Imager strand	Imager conc. (nM)	Frames	Power density (kW cm^{-2})	Integration time (ms)
P1	2.5	80,000	1.24	100
P3 (9EG7)	2.5 (1)	80,000	1.24	100
P3 qPAINT	2.5	160,000	0.82	100
R1	0.25	80,000	1.24	100
R2	0.25	80,000	1.24	100

Supplementary Table 5 | DBSCAN and filtering parameter.

**Imager strand**	**1. DBSCAN [*ε* (px); MinPts]**	**Mean frame**	**2. DBSCAN [*ε* (px); MinPts]**	**Std frame**	**3. DBSCAN [*ε* (px); MinPts]**
P1	0.08; 30	mean ± std	0.08; 30	5000 - 50000	0.05; 20
P3	0.08; 15	mean ± std	0.08; 15	5000 - 50000	0.05; 20
R1	0.08; 15	mean ± std	0.08; 15	5000 - 50000	0.06; 15
R2	0.08; 15	mean ± std	0.08; 15	5000 - 50000	0.06; 15

Supplementary Table 6 | Parameter for DNA-PAINT simulations.

Parameter	Value
k_{on}	1715000 $M^{-1}s^{-1}$
Imager concentration	2.5 nM
Bright time	400 ms
Incorporation	100 %
Power density	1.24 kW cm^{-2}
Photon detection rate	30 Photons ms^{-1} kW^{-1} cm^{2}
Integration time	100 ms
Frames	80,000
Pixel size	130 nm

Supplementary Table 7 | Sample size and experimental repeats.

Figure Notation	Batch Name	Experimental Days (N)	Sample Number (n)
Figure 1i	FA & Origami	3	8 (4/2/2)
Figure 1l/m	1xP3 & 3xP3	1	1
Figure 1o	single & dual	1	1
Figure 2b	5min	3	11 (6/2/3)
	15min	3	9 (1/3/5)
	25min	3	7 (3/1/3)
	40min	4	10 (3/2/2/3)
	16hrs	3	8 (4/2/2)
Figure 2c	FA & MEM	3	13 (5/5/3)
Figure 2e	Random Sim	1	8
Figure 2f	Dimer Sim	1	4
Figure 2g	Molecular Ruler Sim	1	4
Figure 2h	Distribution	3	8 (4/2/2)
Figure 2i	Exp	12	39 (4/4/3/2/2/5/4/3/3/3/1/5)
Figure 3c/d	K2K & T2T & K2T	4	17 (4/3/7/3)
Figure 3e	S1	1	6
	c	3	15 (5/6/4)
	i	4	17 (4/3/7/3)
	CalC	3	15 (5/5/5)
	S2	1	5
Figure 3h	all sim points	1	4
Figure 4d/e	I2I &K2K &T2T & I2K & I2T	1	6
Supplementary Fig. 2c	NeNa	39	209
Supplementary Fig. 3b	FA & Origami	3	8 (4/2/2)
Supplementary Fig. 3c	FA & Origami	1	1
Supplementary Fig. 4	talin localization clouds	3	1076/947/903

Supplementary Fig. 6d	20 nm	1	4
	25 nm	1	4
	30 nm	1	1
	35 nm	1	1
Supplementary Fig. 7b/c	Origami	11	24
	15’	1	1
	25’	2	4 (3/1)
	40’	3	7 (3/2/2)
	16 h	3	8 (4/2/2)
	CalC	1	3
	3xP3	1	1
Supplementary Fig. 8b/c	Nb & Halo	1	3
Supplementary Fig. 8d/e	1st & 2nd	1	5
Supplementary Fig. 9c/d	P3	5	13 (2/3/4/2/2)
	P1	3	11 (4/3/4)
Supplementary Fig. 9g/h	SNAP-P3 & Halo P1	3	14 (5/4/5)
Supplementary Fig. 10c/d	K2K & T2T & K2T	3	12 (4/5/3)
Supplementary Fig. 10e	S3	1	8
	i	3	12 (4/5/3)
	CalC (as in Fig.3e)	3	15 (5/5/5)
Supplementary Fig. 11a/b	all sim points	1	5
