# Supplementary material for: Quantitative single-protein imaging reveals molecular complex formation of integrin, talin, and kindlin during cell adhesion
Source: Nat Commun. 2021 Feb 10;12:919. doi: 10.1038/s41467-021-21142-2 (PMC7876120; doi:10.1038/s41467-021-21142-2)
Supplement: Supplementary file 3 — Reporting Summary [file 41467_2021_21142_MOESM3_ESM.pdf]

nature research

Corresponding author(s): Reinhard Fässler
Ralf Jungmann
Carsten Grashoff

Last updated by author(s): Jan 11, 2021

Reporting Summary

Nature Research wishes to improve the reproducibility of the work that we publish. This form provides structure for consistency and transparency in reporting. For further information on Nature Research policies, see our Editorial Policies and the Editorial Policy Checklist.

Statistics

For all statistical analyses, confirm that the following items are present in the figure legend, table legend, main text, or Methods section.

n/a	Confirmed	
☐	☒	The exact sample size (*n*) for each experimental group/condition, given as a discrete number and unit of measurement
☐	☒	A statement on whether measurements were taken from distinct samples or whether the same sample was measured repeatedly
☐	☒	The statistical test(s) used AND whether they are one- or two-sided<br>*Only common tests should be described solely by name; describe more complex techniques in the Methods section.*
☒	☐	A description of all covariates tested
☐	☒	A description of any assumptions or corrections, such as tests of normality and adjustment for multiple comparisons
☐	☒	A full description of the statistical parameters including central tendency (e.g. means) or other basic estimates (e.g. regression coefficient) AND variation (e.g. standard deviation) or associated estimates of uncertainty (e.g. confidence intervals)
☐	☒	For null hypothesis testing, the test statistic (e.g. *F*, *t*, *r*) with confidence intervals, effect sizes, degrees of freedom and *P* value noted<br>*Give P values as exact values whenever suitable.*
☒	☐	For Bayesian analysis, information on the choice of priors and Markov chain Monte Carlo settings
☒	☐	For hierarchical and complex designs, identification of the appropriate level for tests and full reporting of outcomes
☒	☐	Estimates of effect sizes (e.g. Cohen's *d*, Pearson's *r*), indicating how they were calculated

Our web collection on statistics for biologists contains articles on many of the points above.

Software and code

Policy information about availability of computer code

Data collection	For DNA-PAINT imaging on Nikon Eclipse Ti microscope (custom-build) the Micro-Manager Open Source Microscopy Software (VersionXY) was used. For DNA-PAINT imaging on a commercially available Nikon Ti-E N-SIM/N-STORM setup the NIS-Elements (Nikon) software (VersionXY) together with a custom-written ImageJ Macro have been used (ImageJ2 https://imagej.net/ImageJ2). All DNA-PAINT images were analyzed using Picasso Software.
Data analysis	DNA-PAINT data was analyzed using Picasso software (v0.3.0, available on Github) and custom-written python scripts (Version 3.7) and ImageJ2 (ImageJ2 https://imagej.net/ImageJ2).<br>Two sided 2D Kolmogorov–Smirnov (KS) statistical test was modified from Github (github.com/syrte/ndtest) and performed in python (Version 3.7).<br>Two-sample t-test analysis and gaussian distribution fits were performed using OriginPro software (Version 2019, Version 2020).

For manuscripts utilizing custom algorithms or software that are central to the research but not yet described in published literature, software must be made available to editors and reviewers. We strongly encourage code deposition in a community repository (e.g. GitHub). See the Nature Research guidelines for submitting code & software for further information.

Data

Policy information about availability of data

All manuscripts must include a data availability statement. This statement should provide the following information, where applicable:

- Accession codes, unique identifiers, or web links for publicly available datasets
- A list of figures that have associated raw data
- A description of any restrictions on data availability

The authors confirm that all relevant data are included in this published article (and its supplementary information files). Additional data is available upon request.

Field-specific reporting

Please select the one below that is the best fit for your research. If you are not sure, read the appropriate sections before making your selection.

- [x] Life sciences
- [ ] Behavioural & social sciences
- [ ] Ecological, evolutionary & environmental sciences

For a reference copy of the document with all sections, see nature.com/documents/nr-reporting-summary-flat.pdf

Life sciences study design

All studies must disclose on these points even when the disclosure is negative.

Sample size	No sample size calculation was performed prior to experiments. In general, samples were collected on three different measurement days for all relevant data to achieve sample sizes of about n=10. Each sample accumulates distance data of minimum several hundred independent protein interdistance measurements in one to three different regions of interest within a given cell resulting in thousands of independent interdistance measurements per sample. If sample size was in the range of n=10, statistical tests such as two sample t-tests were performed to depict tendencies. Backup control experiments were partly measured only once to verify tendencies. The three color superresolved data accumulation was performed once only, standard statistical analyses therefore not performed. However, the accumulated tupled interdistance data were statistically analysed using 2D Kolmogorov-Smirnov test in combination with custom written bootstrapping analysis to verify 2d-statistical differences.
Data exclusions	Data was excluded if NeNa calculation of the sample image was >25 nm. Data were also excluded if faulty imaging conditions (e.g. unstable imager concentration, image alignment errors, or protein expression artefacts) were detected that induced error-prone DBSCAN and thereby false protein interdistance results.
Replication	All relevant experiments were replicated at three different experimental days using newly generated samples. The three-color superresolved data were not replicated on separate experimental days as the data were analysed on the basis of protein interdistance tuples; the 2d distribution of thousands of independent interdistance measurements (data pool n>30.000 tuples) was validated by 2d statistical analysis and bootstrapping.<br>All replication attempts were successful.
Randomization	Samples were measured batch after batch to ensure equivalent measurement properties within the sample batch. Randomization was not relevant for this study except for the bootstrapping analysis. Here n=1000 data tuples out of complete data sets (n>30000) were randomly sampled (repetition number =1000) and statistically compared using custom written two sided 2D Kolmogorov-Smirnov test.
Blinding	Blinding of investigators was not relevant and necessary for this study as data generation could not be performed selectively due to the necessity of image data reconstruction after data generation.

Reporting for specific materials, systems and methods

We require information from authors about some types of materials, experimental systems and methods used in many studies. Here, indicate whether each material, system or method listed is relevant to your study. If you are not sure if a list item applies to your research, read the appropriate section before selecting a response.

Materials & experimental systems

n/a	Involved in the study	
[ ]	[x]	Antibodies
[ ]	[x]	Eukaryotic cell lines
[x]	[ ]	Palaeontology and archaeology
[x]	[ ]	Animals and other organisms
[x]	[ ]	Human research participants
[x]	[ ]	Clinical data
[x]	[ ]	Dual use research of concern

Methods

n/a	Involved in the study	
[x]	[ ]	ChIP-seq
[x]	[ ]	Flow cytometry
[x]	[ ]	MRI-based neuroimaging

Antibodies

Antibodies used	rat anti-Integrin β1 [9EG7] (BD Pharmingen, 550531)<br>GFP Nanobody (NanoTag, Clone 1B2, unconjugated, 0303)
Validation	rat anti-Integrin β1 [Clone 9EG7], pruified rat anti-mouse CD29 (BD Pharmingen, 550531, after conjugation diluted 1:200)<br>immunogen: mouse endothelial cell line<br>reactivity: QC testing mouse<br>applications: Flow cytometry (Routinely Tested), Immunohistochemistry-frozen, Immunohistochemistry-zinc-fixed (Tested During Development), Immunohistochemistry-formalin (antigen retrieval required) (Not Recommended)

Camelid sdAb anti-GFP (NanoTag, Clone 1B2, unconjugated , 0303, after conjugation diluted 1:200)
Specificity: Recognizes GFP, mEGFP, superfolder GFP and most common CFP and YFP variants. Does not cross-react with mCherry, mRFP, dsRed, mTagBFP or their most common derivatives.
applications: immunohistochemistry and immunofluorescence recommended dilution 1:500.

Eukaryotic cell lines

Policy information about cell lines

Cell line source(s)	talin 1/2 double knockout cells: fibroblastoid kidney cells of a three week-old mouse were generated by transducing SV40 large T immortalized tln1(f/f) tln2(−/−) cells with Cre recombinase and isolating a clonal cell line (Theodosiou et al, Elife, 2016).<br>quadruple knockout fibroblasts deficient for talin-1, talin-2, kindlin-1 and kindlin-2 (Tln1-/-Tln2-/- K1-/-K2-/-; qKO) were isolated from mice carrying floxed kindlin-1 (Fermt1flox/flox), kindlin-2 (Fermt2flox/flox), Tln1 alleles, and nullizygous Tln2 alleles. The floxed alleles were deleted by adenoviral expression of Cre recombinase, resulting in kindlin-1, kindlin-2, talin-1, and talin-2–deficient (quadruple knockout (qKO)) cells (Böttcher et al, JCB, 2017).
Authentication	no authentication
Mycoplasma contamination	Cell lines were not tested for mycoplasm contamination.
Commonly misidentified lines<br>(See ICLAC register)	No commonly misidentified cell line was used.
